# Measurement-guided therapeutic-dose prediction using multi-level gated modality-fusion model for volumetric-modulated arc radiotherapy

**DOI:** 10.3389/fonc.2025.1468232

**Published:** 2025-03-19

**Authors:** Changfei Gong, Yuling Huang, Junming Jian, Wenheng Zheng, Xiaoping Wang, Shenggou Ding, Yun Zhang

**Affiliations:** 1 Department of Radiation Oncology, Jiangxi Cancer Hospital & Institute, Nanchang, Jiangxi, China; 2 Jiangxi Clinical Research Center for Cancer, Nanchang, Jiangxi, China; 3 NHC Key Laboratory of Personalized Diagnosis and Treatment of Nasopharyngeal Carcinoma (Jiangxi Cancer Hospital), Nanchang, Jiangxi, China

**Keywords:** artificial intelligence, radiotherapy, therapeutic-dose prediction, pre-treatment patient-specific quality assurance, multi-level gated modality fusion network

## Abstract

**Objectives:**

Radiotherapy is a fundamental cancer treatment method, and pre-treatment patient-specific quality assurance (prePSQA) plays a crucial role in ensuring dose accuracy and patient safety. Artificial intelligence model for measurement-free prePSQA have been investigated over the last few years. While these models stack successive pooling layers to carry out sequential learning, directly splice together different modalities along channel dimensions and feed them into shared encoder-decoder network, which greatly reduces the anatomical features specific to different modalities. Furthermore, the existing models simply take advantage of low-dimensional dosimetry information, meaning that the spatial features about the complex dose distribution may be lost and limiting the predictive power of the models. The purpose of this study is to develop a novel deep learning model for measurement-guided therapeutic-dose (MDose) prediction from head and neck cancer radiotherapy data.

**Methods:**

The enrolled 310 patients underwent volumetric-modulated arc radiotherapy (VMAT) were randomly divided into the training set (186 cases, 60%), validation set (62 cases, 20%), and test set (62 cases, 20%). The effective prediction model explicitly integrates the multi-scale features that are specific to CT and dose images, takes into account the useful spatial dose information and fully exploits the mutual promotion within the different modalities. It enables medical physicists to analyze the detailed locations of spatial dose differences and to simultaneously generate clinically applicable dose-volume histograms (DVHs) metrics and gamma passing rate (GPR) outcomes.

**Results:**

The proposed model achieved better performance of MDose prediction, and dosimetric congruence of DVHs, GPR with the ground truth compared with several state-of-the-art models. Quantitative experimental predictions show that the proposed model achieved the lowest values for the mean absolute error (37.99) and root mean square error (4.916), and the highest values for the peak signal-to-noise ratio (52.622), structural similarity (0.986) and universal quality index (0.932). The predicted dose values of all voxels were within 6 Gy in the dose difference maps, except for the areas near the skin or thermoplastic mask indentation boundaries.

**Conclusions:**

We have developed a feasible MDose prediction model that could potentially improve the efficiency and accuracy of prePSQA for head and neck cancer radiotherapy, providing a boost for clinical adaptive radiotherapy.

## 1 Introduction

Cancer remains the leading cause of mortality worldwide, and has threatened human life with increasing rates of incidence over the past few years (1, 2). Along with surgery, chemotherapy and immunotherapy, radiotherapy (RT) is a crucial pillar of cancer treatment that destroys the target cells with ionizing radiation and deprives them of the ability to divide and proliferate, while sparing the surrounding healthy tissues (3). Previous and emerging innovations in hardware and software have contributed to the creation and delivery of the advanced volumetric-modulated arc radiotherapy (VMAT) technique, which yields significant improvements in terms of target coverage, sparing of organs at risk (OARs), and treatment efficiency through dynamic MLC modulation based on variable rotary gantry angles (4, 5). However, the extensive modulations utilized in the highly conformal approach often led to increased complexity and dosimetric error in the dose calculation or delivery system. Pre-treatment patient-specific quality assurance (prePSQA) is an indispensable clinical process which plays a crucial role in ensuring dose accuracy and patient safety, as strongly recommended by the American Association of Physicists (6). One conventional method of prePSQA is to measure the transmitted dose distribution using radiographic film, diode/ion chamber array or electronic portal imaging device (EPID) (7, 8). Nevertheless, the execution of measurement-based prePSQA is expensive and time-consuming for busy radiation oncology centers. Only a very small percentage of treatment plans will fail these checks, and yet all of them are carried out, creating an unnecessary burden for medical physicists. If prePSQA fails, reformulating the plan will disrupt the conventional clinical workflow and defer treatment, often causing confusion and frustration to cancer patients (9).

### 1.1 Traditional Methods

In recent years, data-driven artificial intelligence (AI) has made tremendous developments in computer vision, natural language processing and medicine (10–12). In this paper, we mainly review the AI-based prePSQA literatures. The application of machine learning (ML) to measurement-free prePSQA has been achieved through traditional approaches over the past ten years. With a focus on studies of gamma passing rate (GPR) prediction models, Valdes et al. were the first to use a Poisson regression with the Lasso regularization method for predicting “pass” or “fail” (PRF) for intensity modulated radiation therapy with a gamma evaluation criterion of 3%(local)/3 mm (13). They reported results that demonstrated a strong or moderate linear relationship between the predicted and measured values, and especially for the detection of failures due to incorrect settings and miniscule differences between matched LINACs. Later, they performed a multi-center study to validate the virtual QA approach using different measurement devices on multiple LINACs (14). In a similar study, Li et al. used a regression model to predict individual GPRs and a classification model to classify PRF for VMAT plans (15). By exploring the applicability of artificial neural networks (ANN) to the field of dosimetry, Chan et al. demonstrated that ANN time-series modeling had the advantage of more accurate and effective prediction over the well-developed auto-regressive moving average technique on long-term accumulated datasets (16). Hirashima et al. used an ML technique to improve the prediction and classification performance for GPRs with plan complexity and dosimetric features (17). Granville et al. also trained a linear support vector machine to classify the metrics of VMAT prePSQA measurement by using both plan characteristics and routine quality control results (18). Although these automatic methods have improved the efficiency of prePSQA to some extent, and have potential benefits in terms of solving some issues, their prediction accuracy is not high and they rely heavily on the manual extraction/learning of complex features, resulting in poor scope for clinical applications.

### 1.2 Deep Learning Models

In deep learning (DL) with convolutional neural networks (CNN), multi-layer features are abstracted automatically and integrated into an end-to-end network for prediction, which contributes to eliminating the dependence on handcrafted features. Recently, many DL-based models have shown success when applied to image segmentation, and have been introduced to the field of prePSQA. For example, Tomori et al. developed a 15-layer CNN-based prediction model for prePSQA in prostate cancer treatment (19). This prediction model was later improved by using dummy target plans and predicted GPR values in various gamma criteria (20). By using transfer learning from rotation and translation of the fluence maps during training, Interian et al. created the convolutional blocks of a VGG-16 ImageNet and compared it to a generalized Poisson regression model (21). They found that deeply fine-tuned CNNs outperformed a baseline system designed by domain experts when applied to the prediction of 3%/3mm local GPRs. Kadoya et al. studied a DL-based prediction model for gamma evaluation and applied it to prostate cancer cases (22). Hu et al. proposed an automatic multi-branch neural network model based on metrics describing the complexity of RT, and proved that this model could assist physicians to improve QA in terms of efficiency and quality (23). Rather than directly predicting GPRs for IMRT/VMAT plans from complexity metrics derived from the physical characteristics of the plans and machine-related parameters, Nyflot et al. built a CNN model to classify the presence or absence of introduced delivery errors generated from fluence maps and measured by EPID, and demonstrated that the performance of their network was superior to a handcrafted approach with texture features (24). Jia et al. reported a novel GAN architecture to carry out the EPID image-to-dose conversion, and their results showed that a DL-based signal processing strategy could accurately predict the cylindrical phantom dose (25). Other studies have investigated improved DL networks to model GPR predictions by using delivery fluence distribution informed by log files (26–28).

### 1.3 Limitations

Previous findings have demonstrated the potential and feasibility of ML/DL models in terms of predicting prePSQA without performing real measurements. However, in the light of these studies (11–29), two major issues should be considered when building a ML/DL prePSQA model in clinical settings. Firstly, these methods predicted prePSQA or to identify protentional dose errors by inputting planar dose, fluence maps, or treatment plan/LINAC performance-related metrics (29). The low-dimensional radiotherapy data are deficient in showing dose correlation between adjacent layers, making it impossible to explore the spatial features of the volumetric dose and resulting in limited sensitivity to detect clinical dose errors. Currently, evaluation of the dose-volume histograms (DVHs) between the unapproved RT dose and the measurement-reconstructed patient volumetric dose (MDose) has been incorporated into clinical practice. The MDose can provide useful spatial information about the complex dose distribution, especially for VMAT cases. From the distribution of MDose, dosimetric metrics for all structures can be completely reconstructed and detailed spatial dose differences can be displayed (30). Secondly, the most existing prePSQA prediction networks stack successive convolutional and pooling layers to obtain robust feature representations, and directly splice together different modalities along channel dimensions and feed them into one shared encoder-decoder network, which greatly reduces spatial feature resolution specific to different modalities and may lead to dose prediction errors for small structures. Our previous study falls into this category, reporting the feasibility of a ResUNet model and a ML model for predicting the metrics of prePSQA (31).

### 1.4 Contributions

In this work, we overcome the two major issues outlined above by designing a new prePSQA prediction approach for head-and-neck (H&N) patients receiving VMAT, which enables physicists to analyze the detailed locations of spatial dose differences and has the potential to improve the accuracy and efficiency of prePSQA. The contributions of this study are summarized as follows:

1. We propose a novel deep network model for prePSQA to acquire high-quality voxel-wise MDose. Instead of predicting prePSQA metrics derived from the physical characteristics of the plans and machine-related parameters, we provide the MDose-based prePSQA model that receive the volumetric RT dose (RTDose) and CT images as input, and then directly output the patient-specific predicted volumetric. This design has the benefits of integrating robust global semantic information with local spatial details for MDose prediction, and simultaneously generating clinically applicable DVHs metrics and GPR outcomes.
2. We design a novel multi-level gated modality fusion architecture (MLGMF) that employs different encoder sub-networks to extract the multi-scale features that are specific to CT and RTDose. By introducing the MLGMF into the squeeze-and-excitation residual connection mechanism-based CNN, the proposed model fully exploits the mutual promotion within different modalities, and explicitly integrates useful contextual information with rich spatial details for MDose prediction.
3. We demonstrate in the extensive experiments that our method can achieve comparable or better performance on MDose prediction, DVHs metrics and estimation of GPR compared to the existing state-of-the-art methods. Furthermore, the present MDose-based prediction approach greatly improves the efficiency for prePSQA with a practical solution and is a promising direction for clinical adaptive RT.

## 2 Materials and methods

### 2.1 Patient characteristics

A total of 310 patients with H&N cancer treated with VMAT between 2018 and 2022 were enrolled in this study. **Table 1** summarizes the clinical characteristics of these patients. All the patients were immobilized with a thermoplastic mask in the supine/prone position, computed tomography, magnetic resonance imaging and positron emission tomography images were used by experienced experts to help contour the target volumes according to international guideline. The prescribed doses were 70 and 56 Gy or 70, 63, and 56 Gy in 35 fractions. VMAT plans were generated by an experienced physicist to achieve clinically acceptable target volume coverage while sparing OARs. This was done using Pinnacle treatment planning system (TPS) ver. 9.10 with the SmartArc optimization algorithm or Monaco TPS ver. 5.11 with the Monte Carlo algorithm, and executed on an Elekta Infinity equipped with an Agility MLC or a Varian TrueBeam equipped with a Millennium 120 MLC.

**Table 1:** Summary of the enrolled patients.

Characteristics	Training cohort (186)	Validation cohort (62)	Testing cohort (62)
Gender, no. (%)			
Male	101 (54.3%)	45 (72.6%)	42 (67.7%)
Female	85 (45.7%)	17 (27.4%)	20 (32.3%)
**Age (years)**	58.3 ± 14.3	54.1 ± 18.8	55.2 ± 17.4
<20y	6 (3.2%)	4 (6.5%)	3 (4.8%)
20y-40y	28 (15.1%)	9 (14.5%)	11 (17.7%)
40y-60y	67 (36.0%)	18 (29.0%)	20 (32.3%)
>60y	85 (45.7%)	31 (50%)	28 (45.2%)
Pathological type			
SCCA	65 (34.9%)	23 (37.1%)	19 (30.6%)
LA	34 (18.3%)	16 (25.8%)	13 (21.0%)
SA	28 (15.1%)	9 (14.5%)	6 (9.7%)
ACCA	26 (14.0%)	10 (16.1%)	14 (22.6%)
MCA	33 (17.7%)	4 (6.5%)	6 (9.7%)

SCCA, Squamous cell carcinoma; ACCA, Adenoid cystic carcinoma; MCA, mucoepidermoid carcinoma; SA, Sarcoma; LA, Lymphoma.

### 2.2 Measurement and data collection

In this work, dose distribution was measured using only one validation device per patient, before patient plan validation, we performed common physics checks on the LINACs and TPS modeling to ensure that they were in normal condition. Then prePSQA for each VMAT plan was performed by two 3D dose-verification systems (VerSys). One is a Dolphin-Compass online treatment monitoring system ver. 3.0 (IBA Dosimetry, Schwarzenbruck, Germany), which included an array of 1,513 air vented ionization chambers with a spatial resolution of 0.5 cm in the central area. The wireless Dolphin transmission detector was mounted and secured on a LINAC gantry head for measurements, and was optimized for rotational treatments with a built-in gantry angle sensor. Dose reconstruction software from Compass was used to verify the plan, the beams, and the control segments of the VMAT arcs. Patient-specific MDose reconstruction for each patient’s anatomy was performed based on fluence measurements of a phantom and an advanced collapsed cone convolution superposition algorithm on the planning CT. The other is an ArcCHECK system (Sun Nuclear Corporation, Melbourne, FL, USA). A strict calibration procedure of the two systems, including verification the accuracy of array measurement, dose reconstruction and beam modeling, was performed in advance according to the manufacturer’s standards. In essence, the aim of this method was to use the dose distribution measured inside a QA phantom with a relatively low pixel density detector array to guide the TPS dose for the patient dataset, resulting in a high voxel-density MDose distribution. We also recorded and evaluated the percentage dosimetry errors, volumetric error and GPRs between the planned and reconstructed doses, where GPRs were calculated using a 3%/3mm criterion with a 10% threshold, and a GPR value of 90% was employed to determine pass or fail. To ensure target coverage, the values of Dmean and Dmax (mean and maximum doses), D2, D98, V95 and V100 (where Dx means the dose received by x% of the volume, and Vx represents the volume receiving at least x% of the prescription dose) for the targets were analyzed. For the assessment of OARs, Dmax values of brainstem, bilateral lens, optic chiasm, spinal cord, pituitary, optic nerves and temporomandibular joints were evaluated, and Dmean values of D50 for the bilateral parotid were also calculated.

### 2.3 Flow of this study

**Figure 1:**
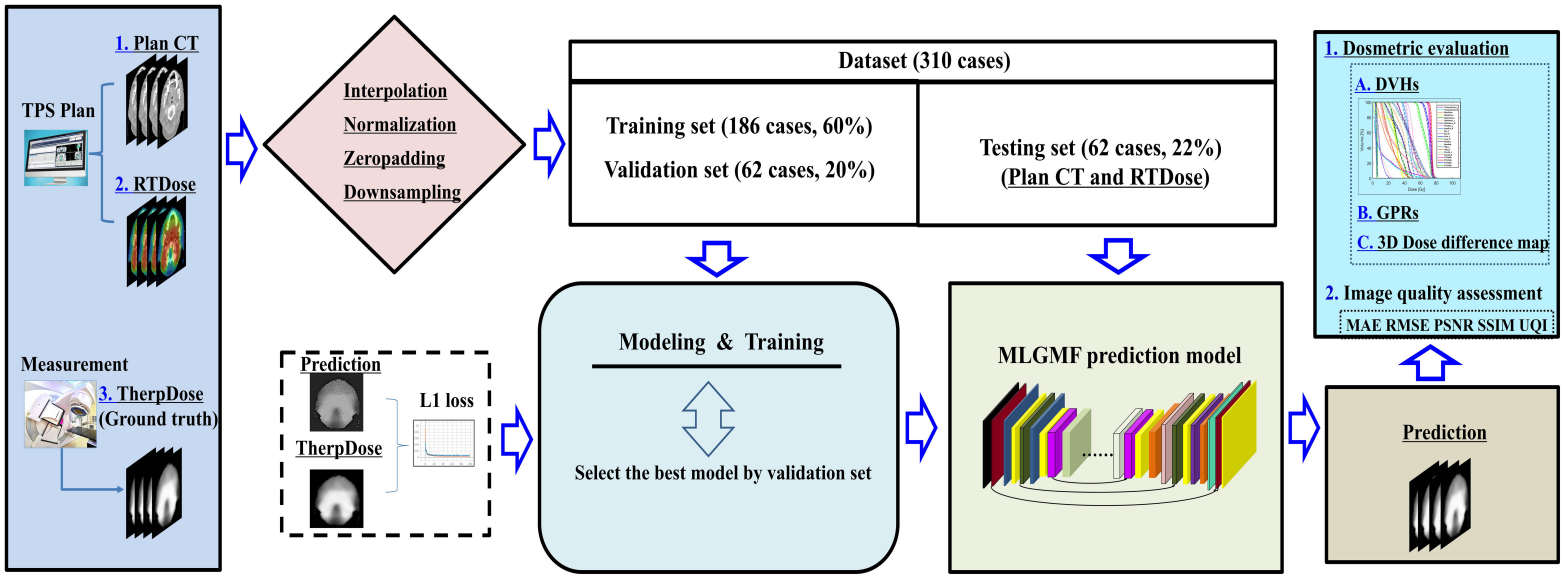
The overall workflow of this study.

**Figure 1** illustrates the overall workflow of our method. Firstly, we extracted the DICOM files of CT and RTDose for the VMATs, following the procedure for dosimetric measurement for each patient using 3D VerSys. Then, a U-Net-like baseline model and four specially designed MLGMF-based networks were trained to predict the voxel-level MDose distribution. These five prediction models took CT slices and the RTDose as input, as well as the MDose distribution from the VerSys, and output the predicted MDose distribution of the corresponding slice. Here, we referred the predicted MDose as PDose. Finally, an in-house Python code was developed to calculate the relevant DVHs metrics, 3D dose difference maps, and GPRs from the PDose for each specific patient.

### 2.4 Baseline model

The task of MDose prediction can be formulated as a dense voxel-level prediction problem. Low-level spatial details and high-level semantic cues are both essential for this challenging problem. In view of this, effective fusion of the multi-level features is the key to obtaining an accurate prediction result. The widely used U-Net architecture employs an encoder-decoder model to combine these multi-scale features. According to the findings of our previous study in which the widely used U-Net encoder-decoder architecture was used as the main network (31). However, since the CT and RTDose for each patient are both in 3D form, we changed the building block of U-Net to a 3D paradigm and introduced a squeeze-and-excitation residual connection mechanism, thus forming the baseline model of ResUNet.

### 2.5 The proposed MLGMF

Due to the difference in modality between the CT and RTDose, it is not sufficient to simply concatenate the CT and RTDose as the input to the encoder-decoder network. In order to fully exploit the mutual promotion within the two different modalities, we propose a multi-level modality fusion architecture (MLGMF). Specifically, two different encoder sub-networks are employed to extract the multi-scale features that are specific to the CT and RTDose modalities. Following this, a single shared decode sub-network fuses the multi-scale features generated by the CT and RTDose encoder sub-networks in a layer-wise manner. Similarly to the original ResUNet, the shared decoder sub-network consists of multiple fusion operations, which are employed to fuse the multi-scale features generated by the encoder sub-network in a progressive way. In contrast, features from both the CT branch and RTDose branch are required to be fused by each fusion module in our proposed multi-scale gated modality fusion modules. To achieve this, in the fusion module in the *i*-th layer, the feature maps of the CT branch ( $\bar{f_{i}^{CT}}$) and RTDose branch ( $\bar{f_{i}^{RTDose}}$) are first fused by concatenation along the channel dimension, and another convolution is applied for further feature abstraction. The obtained feature ( $\hat{F_{i}}$) is then fused with the upsampled feature map from the deeper layer $F_{i+1}$, using a fusion pipeline similar to that in U-Net, to generate the final fused feature $F_{i}$ in the *i*-th layer. This operation can be formulated as Equation 1:

(1) $$F_{i}=Conv\left(Concat\left(Up\left(F_{i+1}\right),\;Conv\left(Concat\left(\bar{f_{i}^{CT}},\;\bar{f_{i}^{RTDose}}\right)\right)\right)\right)$$

**Figure 2:**
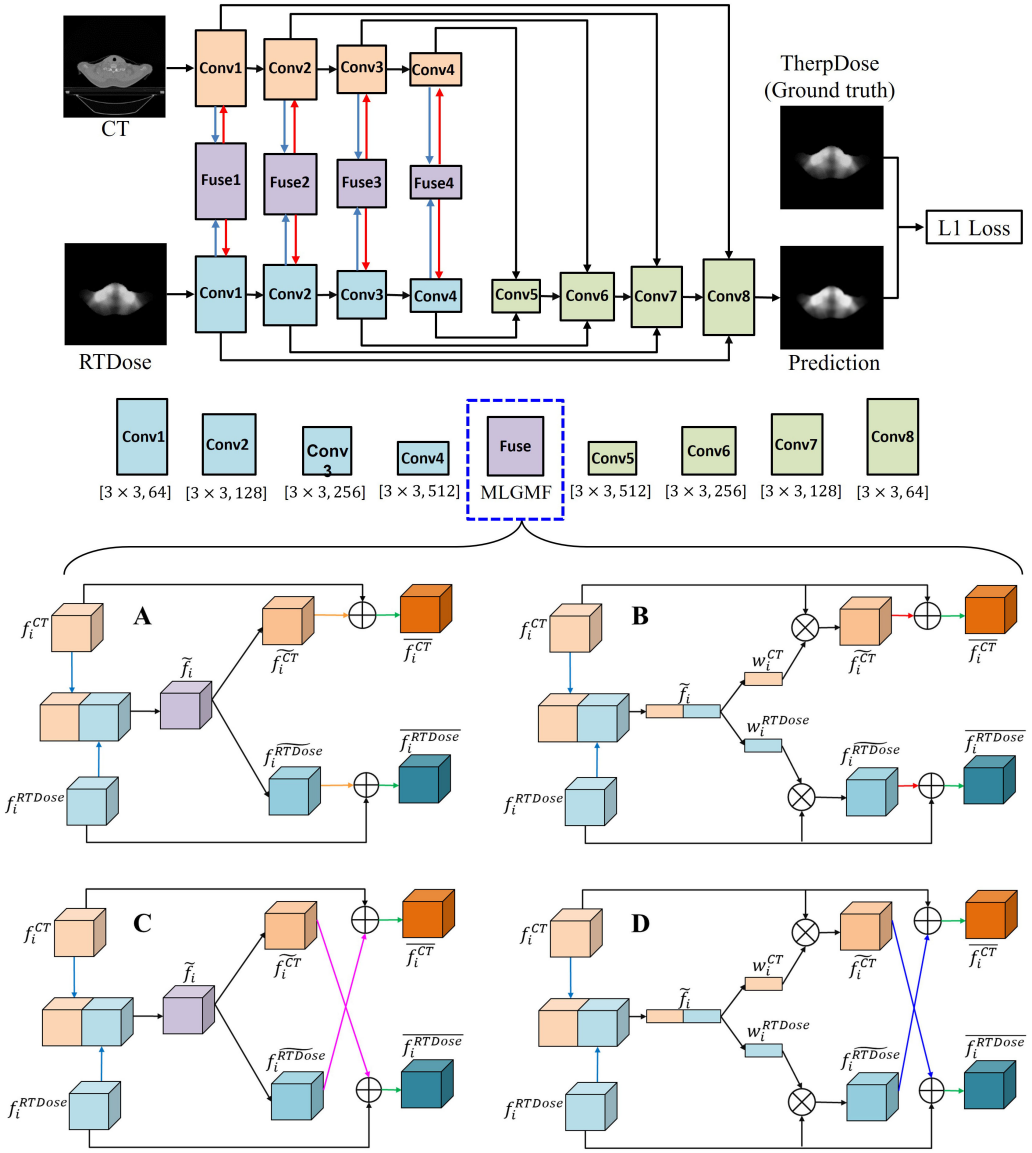
The proposed MLGMF architecture. **(A)** The architecture of the gated modality concatenation parallel fusion module; **(B)** The architecture of the gated modality concatenation cross fusion module; **(C)** The architecture of the gated modality squeezed parallel fusion module; **(D)** The architecture of the gated modality squeezed cross fusion module.

**Figure 3:**
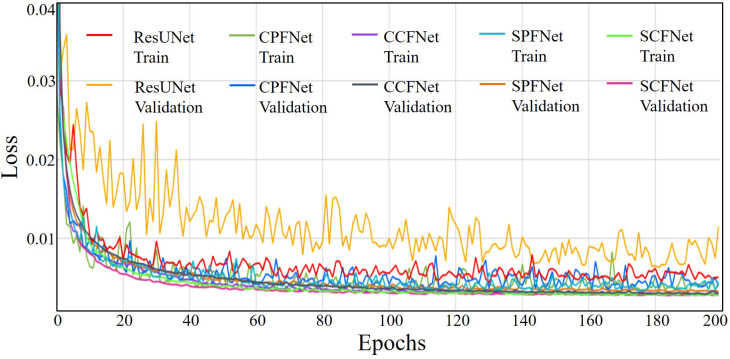
The loss curves of the training and validating process from five methods.

**Figure 4:**
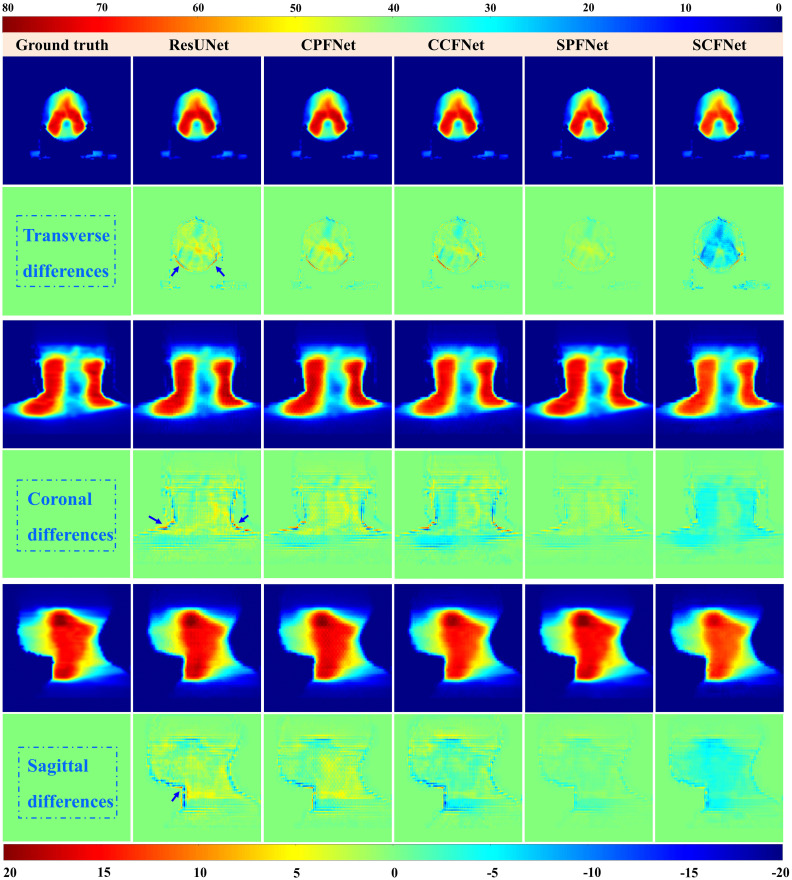
Dose distributions in transverse, coronal and sagittal CT slides from ground truth and five different approaches. The first, third and fifth rows represent the transverse, coronal and sagittal dose distribution, respectively. The second, fourth and last rows illustrate the differences between the ground truth and the predictions.

The fusion operation is iterated until the lowest layer is reached, where the generated feature $F_{1}$ has the same spatial resolution as the input image, which is used to produce the final prediction. The multi-level modality fusion module only fuses the features of CT and RTDose in the decoder module, via simple feature concatenation; however, to encourage mutual interaction between these two different modalities, we enforce multi-level fusion operations between CT and RTDose modality in the encoder module as well. As shown in **Figure 2**, four specifically designed fusion modules are applied to effectively combine the multi-level features of the sub-networks from different modalities, i.e., (1) gated modality concatenation parallel fusion network (CPFNet) (**Figure 2A**); (2) gated modality concatenation cross fusion network (CCFNet) (**Figure 2C**); (3) gated modality squeezed parallel fusion network (SPFNet) (**Figure 2B**); (4) gated modality squeezed cross fusion network (SCFNet) (**Figure 2D**). In this study, the same fusion module was used to replace Fuse1-Fuse4.

### 2.6 Architecture of multi-level feature fusion modules

1) Gated modality concatenation parallel fusion network (CPFNet): In the encoder sub-network, in order to combine the features of the CT and RTDose images ( $f_{i}^{CT}$ and $f_{i}^{RTDose}$) in the *i*-th layer, the proposed gated modality concatenation parallel fusion module first makes the features of two modalities interact with each other via simple feature concatenation along the feature channel dimension. This operation can be formulated as $\hat{f_{i}}=Concat\left(f_{i}^{CT},f_{i}^{RTDose}\right)$. Another convolutional layer is then applied for further feature abstraction, giving a fused feature $\tilde{f_{i}}=Conv\left(\hat{f_{i}}\right)$. Following this, in order to generate specific features corresponding to the CT and RTDose modalities, different processing pipelines are followed: a convolution layer is used to transform the feature $\tilde{f_{i}}$ for both the CT and RTDose pipelines, generating $\tilde{f_{i}^{CT}}$ and $\tilde{f_{i}^{RTDose}}$, respectively, and these are then summed with the original feature, i.e., $\bar{f_{i}^{CT}}=f_{i}^{CT}+\tilde{f_{i}^{CT}}$, $\bar{f_{i}^{RTDose}}=f_{i}^{RTDose}+\tilde{f_{i}^{RTDose}}$. The generated features $\bar{f_{i}^{CT}}$ and $\bar{f_{i}^{RTDose}}$ are the final transformed features for the CT and RTDose modalities. The details of CPFNet are illustrated in **Figure 2A**.

2) Gated modality concatenation cross fusion network (CCFNet): In order to further boost the mutual interaction between the CT and RTDose modalities, we replace the gated modality concatenation parallel fusion module with a gated modality concatenation cross fusion module in the CPFNet described above. Most of the other operations of the proposed new module are the same as in the CPFNet. As shown in **Figure 2C**, when generating and $\bar{f_{i}^{RTDose}}$ in th stage, a cross fusion mode is employed, i.e., $\bar{f_{i}^{CT}}=f_{i}^{CT}+\tilde{f_{i}^{RTDose}}$, $\bar{f_{i}^{RTDose}}=f_{i}^{RTDose}+\tilde{f_{i}^{CT}}$. In this way, the mutual advantages between the CT and RTDose modalities are further explored.

3) Gated modality squeezed parallel fusion network (SPFNet): Both the CPFNet and CCFNet fuse the features from the CT and RTDose via simple feature concatenation between the two modalities. Inspired by SE-Net, we squeeze the concatenated feature map into 1D form via global average pooling. In this way, the cues about the original fused feature along the spatial dimension (*w* ×h) are reduced, and the global distribution of channel-wise responses is obtained. Similarly to the original SE-Net, the later excitation stage explicitly models the channel interdependencies within the squeezed feature. In this process, more informative features are selectively enhanced, and less useful features are suppressed. To achieve this, we developed the gated modality squeezed parallel fusion module, as illustrated in **Figure 2B**. In the encoder sub-network, in order to combine the features of the CT and RTDose images ( $f_{i}^{CT}$ and $f_{i}^{RTDose}$) in the *i*-th layer, the proposed module first concatenates the two feature maps along the channel dimension. Then, a 3D global average pooling layer along the channel dimension is applied to obtain a squeezed feature representation $\tilde{f_{i}}$ in 1D form. These operations can be formulated as: $\tilde{f_{i}}=GAP_{c}\left(Concat\left(f_{i}^{CT},\;f_{i}^{RTDose}\right)\right)$, where $GAP_{c}$ means global average pooling along the channel dimension. We then use one fully connected layer to convert the squeezed feature map $\tilde{f_{i}}$ into $w_{i}^{CT}$ and $w_{i}^{RTDose}$, respectively. The generated weight maps $w_{i}^{CT}$ and $w_{i}^{RTDose}$ are used to reweight the original features $f_{i}^{CT}$ and $f_{i}^{RTDose}$ via channel-wise multiplication, resulting in $\tilde{f_{i}^{CT}}$ and $\tilde{f_{i}^{RTDose}}.$ These operations can be formulated as: $\tilde{f_{i}^{CT}}=FC_{CT}\left(\tilde{f_{i}}\right)\;⊗f_{i}^{CT},\;\tilde{f_{i}^{RTDose}}=FC_{RTDose}\left(\tilde{f_{i}}\right)\;⊗f_{i}^{RTDose}$, where ⊗ means channel-wise multiplication. $\tilde{f_{i}^{CT}}$ and $\tilde{f_{i}^{RTDose}}$ are then summed with the original features, as: $\bar{f_{i}^{CT}}=f_{i}^{CT}+\tilde{f_{i}^{CT}}$, $\bar{f_{i}^{RTDose}}=f_{i}^{RTDose}+\tilde{f_{i}^{RTDose}}$. The generated features $\bar{f_{i}^{CT}}$ and $\bar{f_{i}^{RTDose}}$ are the final transformed features for the CT and RTDose modalities.

4) Gated modality squeezed cross fusion network (SCFNet): Based on the SPFNet model described above, we further enhance the mutual interaction between the CT and RTDose modalities by replacing the gated modality squeezed parallel fusion module with a gated modality squeezed cross fusion module. As shown in **Figure 2D**, most of the operations of the present module are the same as in the SPFNet, and the difference lies in the final feature fusion operation when obtaining $\bar{f_{i}^{CT}}$ and $\bar{f_{i}^{RTDose}}$: $\bar{f_{i}^{CT}}=f_{i}^{CT}+\tilde{f_{i}^{RTDose}}$, $\bar{f_{i}^{RTDose}}=f_{i}^{RTDose}+\tilde{f_{i}^{CT}}$. This cross-fusion mode encourages further mutual interactions between the CT and RTDose branches.

Pseudocode for our implementation of the present algorithm for MDose reconstruction is given in **Algorithm 1**.

**Algorithm 1:**
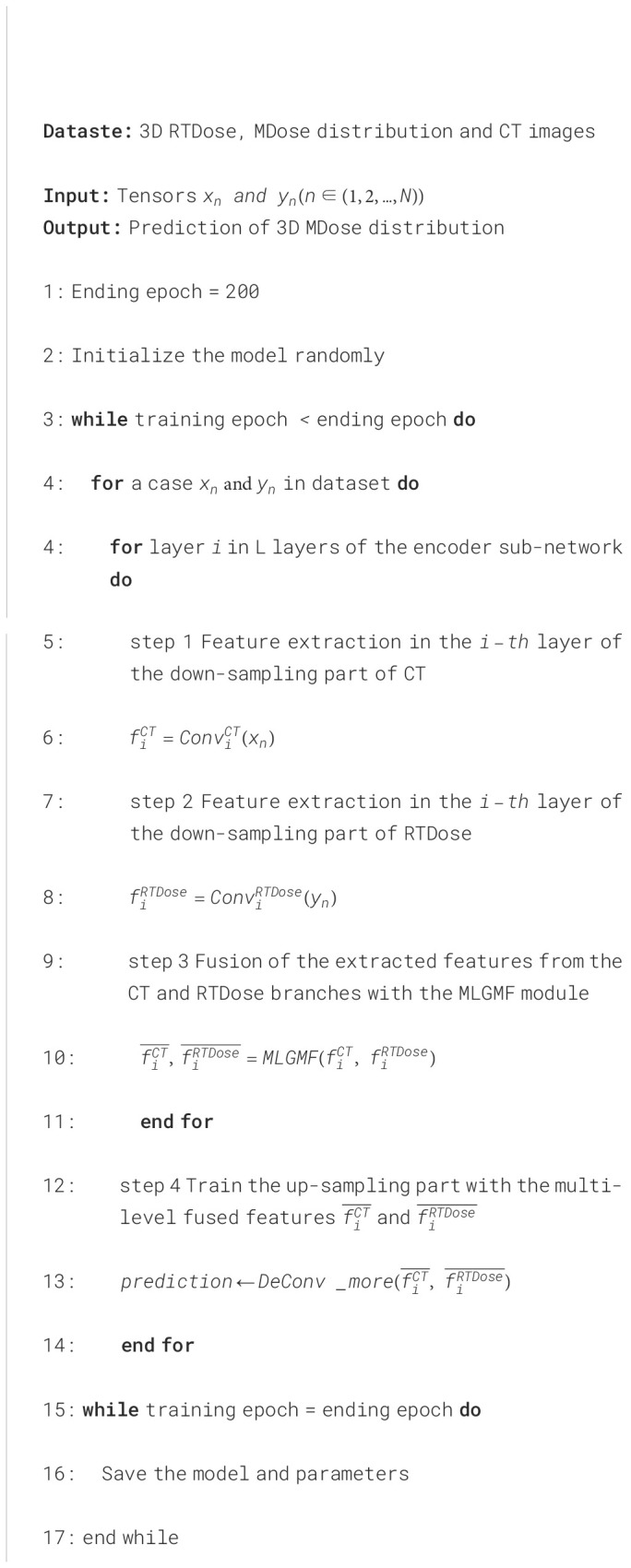
Multi-level Gated Modality Fusion Network.

## 3 Experiments settings

### 3.1 Data preprocessing

In order to reduce the variance of different datasets, calculation complexity, and improve the learning speed, we devised some unified and standardized data processing methods, as well as improved data discretization and attribute selection measures. The volume images were extracted as a 3D matrix with dimensions of 512×512×150. Since the dose distribution depends on the spatial distances among the delineated structures, according to our previous work, (31) the grid resolution for all MDose and RTDose was interpolated to the same pixel size with the corresponding CT coordinates, and zero padding was added during the interpolation process to keep the image size. The specific dosimetry statistics were created using the same dose grid spacing according to the TPS, and were binned as “in or out” for the interest areas using a 3DVH-indices calculation module. If the dose voxel was inside the reconstructed area volume, its dose value was binned; otherwise, it was not counted in the DVH statistics for that area. ^31^ The annotations created by different doctors were mapped to a unified format. To avoid any potential pitfalls such as inflating the testing or validation performance, we were also careful to confirm that the datasets described above were non-overlapping.

### 3.2 Competing methods

To validate and evaluate the performance of MLGMF, several state-of-the-art approaches were considered for comparison purposes, including the ResUNet (29), Swin-UNet (32), self-attention mechanism-based Transformer (TransQA) (31), and invertible and variable augmented network (IVPSQA) (30). ResUNet, this network has recently been used for the prediction of dose distribution, whose input data includes CT, structure, and RTDose derived from TPS, as well as dose distributions measured by the verification system (29). Swin-UNet, first proposed by Cao et al. (32), whom combined the Transformer module and U-shape for medical image segmentation. TransQA was first proposed by Zeng et al. (31), and combines the self-attention mechanism-based Transformer and modified ResUNet for predicting volumetric dose of prePSQA. IVPSQA is derived from the invertible neural networks, Zou et al. (30), first used a modified invertible and variable augmented network to predict the prePSQA metrics from 300 cancer patients who underwent VMAT. These different networks were trained on the same training and validation datasets in the same environment; the maximum number of training epochs was set to 200 for all models, and the learning decay rate was the same for MLGMF. All networks were implemented using the Pytorch library, and we ran the experiments on the same data as for MLGMF. For authenticity, all methods considered for comparison were used with their official open-source codes, and were optimally tuned for the current dataset.

### 3.3 Model training and evaluation

Patients were randomly assigned to the training set (186 cases, 60%), validation set (62 cases, 20%), and test set (62 cases, 20%). The training set was used to enable us to tune the free parameters of the model, the validation set was used to choose the best-performing model, and the test set was used to qualitatively and quantitatively evaluate the performance of the model. Regardless of which method of prediction was used in this study, the grouping of the data was consistent. We employed PyTorch as the backend in our implementation, and the code was run on an NVIDIA GeForce RTX 2080 GPU with 16GB memory and CUDA acceleration. We initialized all the layers of the proposed model with a normalized distribution. Standard backpropagation and an RMSprop optimizer were used to train the model. The learning rate and batch size hyperparameters were set to 5e-5 and one, respectively, and the maximum number of epochs was set to 200. The *L1* loss was applied to supervise the training of the baseline method and our proposed models, which can be formulated as Equation 2:

(2) $$L_{1}\left(Y,\;Y^{ref}\right)=\frac{1}{S\times H\times W}\sum_{s=1}^{S}\sum_{i=1}^{H}\sum_{j=1}^{W}{\left|y_{s,i,j}-y_{\;s,i,j}^{ref}\right|}_{1}$$

where *Y* denotes the PDose, and $Y^{ref}$ denotes the MDose (ground truth: GT). S, H, and W are the slice number, height and width of the specific volume, respectively, and $y_{s,i,j}$ and ${\hat{y}}_{\text{s},\text{i},\text{j}}$ are the predicted value for the (i,j) position of the s-th slice of the prediction and the GT. With the trained prePSQA prediction model, which required about 26 hours of computation, the PDose distribution for a new case with pretreatment RT data took only a few seconds.

To quantitatively evaluate the performance and stability of our method, we used the mean absolute error (MAE), root mean square error (RMSE), peak signal-to-noise ratio (PSNR), structural similarity (SSIM) and universal quality index (UQI) as the evaluation metrics. The MAE and RMSE are accuracy metrics, and were applied to evaluate the predictions of the baseline method and our proposed models. The PSNR, SSIM and UQI were used as indicators of the image quality for the prediction. Mathematically, the detailed calculation of RMSE, MAE, SSIM and UQI can be referred to our previous work (29–31).

## 4 Results

### 4.1 Ablation studies for different feature fusion modules

In this work, we conducted a set of ablation experiments to understand the behavior of the proposed MLGMF and validate its performance of the four embedded modules, i.e., CPFNet, CCFNet, SPFNet, and SCFNet. From training and validating these models, the loss curves in **Figure 3** were obtained. As the number of epochs increased, the loss values for the process of training and validation decreased. After 120-130 epochs, the loss values of training and validation for the proposed models converged to a stable level, showing that further increasing the training epochs should not improve the accuracy of the prediction models.

**Figure 5:**
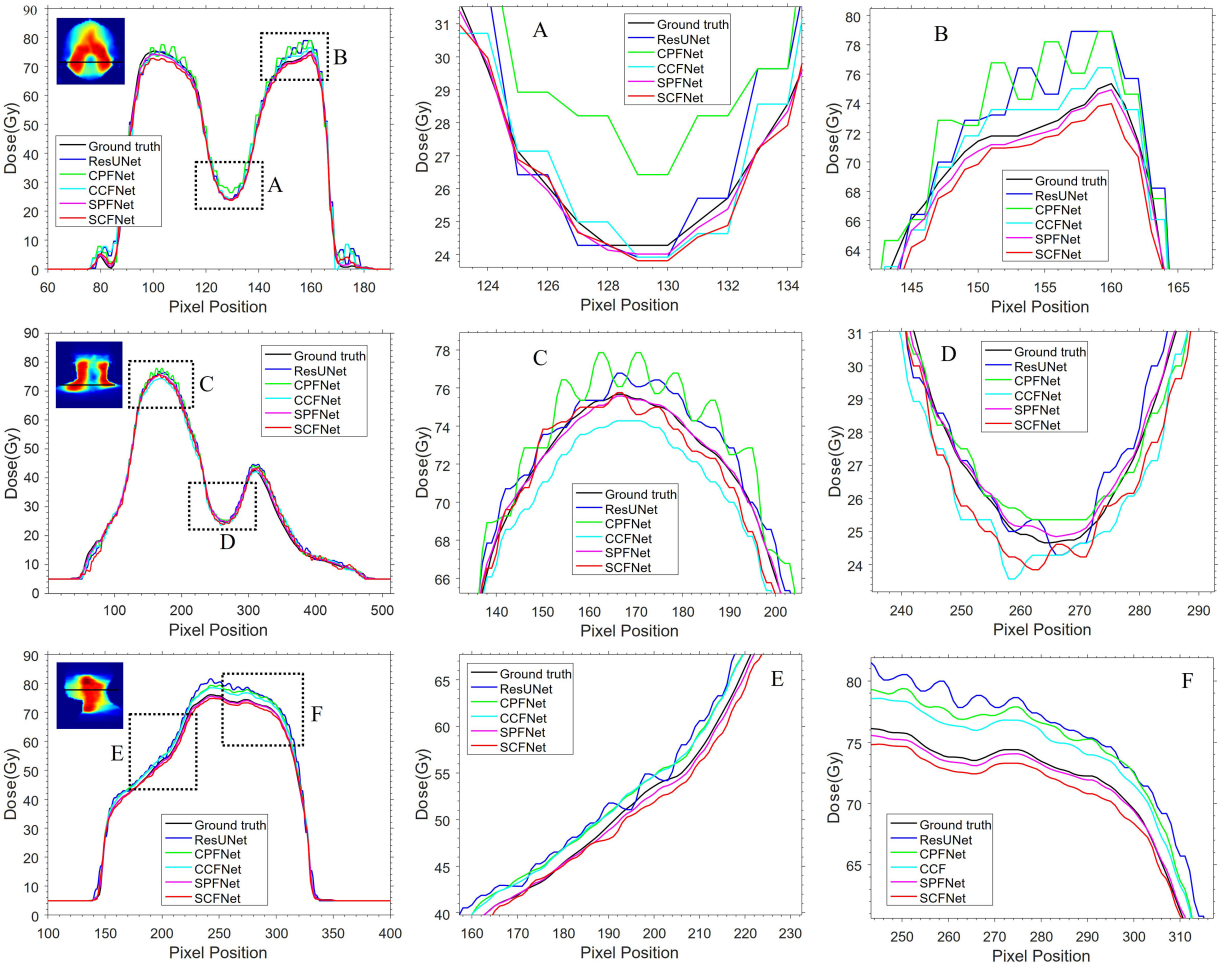
The horizontal difference profiles from transverse, coronal and sagittal dose distribution maps. The second and third columns are the local zoomed-in views of the dashed boxes in first column. The “black line” is from the MDose acts as the ground-truth for comparison, the “blue line”, “green line”, “cyan line”, “magenta line” and “red line” is from ResUNet model, CPFNet, CCFNet, SPFNet and SCFNet, respectively.

Dose distributions for the transverse, coronal, and sagittal slides in the GT images and the five different models are shown in **Figure 4**. We can observe that the four MLGMF-based models achieved better dosimetric congruence with the GT compared with the ResUNet model, and this result was consistent with the study of the dose difference maps. The dose values of all voxels were within 6 Gy in the dose difference maps, except for the areas near the skin or thermoplastic mask indentation boundaries, as indicted by the blue arrows in **Figure 4**. From a comparison of the difference maps among the five methods, smaller dose differences were observed for the four proposed models. SPFNet gave the lowest values of the dose difference, implying that it was able to obtain a better image similarity with the GT. To further evaluate the dose difference, horizontal profiles of the resulting dose maps were drawn across the blue line labeled in **Figure 5**. By observing the zoomed-in views of the dashed boxes, we see that SPFNet and SCFNet were closer to the GT, thus further demonstrating that the proposed models faithfully follow the target/OAR profiles.

To enable a quantitative evaluation of the performance of the five models, **Table 2** lists the values of the MAE, RMSE, PSNR, SSIM and UQI for the predicted results from 22 test case. All metrics were computed over the whole volume in each case, and the top two predictions are shown in bold. SPFNet achieved the lowest values for the MAE (37.99) and RMSE (4.916), and the highest values for the PSNR (52.622), SSIM (0.986) and UQI (0.932). In general, the quantitative results from the MLGMF models were much better than those of the ResUNet. The values of MAE and RMSE for SPFNet were most improved, followed by PSNR and UQI, and finally SSIM. Compared with the ResUNet, the accuracy was improved by 8–52%, 7–38%, and 5–10% as measured by the MAE, RMSE and UQI of the proposed models.

**Table 2:** Quantitative evaluation of ResUNet and the four modules of MLGMF.

	MAE	RMSE	PSNR	SSIM	UQI
ResUNet	79.476	7.905	49.993	0.962	0.844
CPFNet	73.002	7.384	51.079	0.983	0.882
CCFNet	71.235	7.434	49.658	0.982	0.912
SPFNet	37.990	4.916	52.622	0.986	0.932
SCFNet	46.068	5.586	49.153	0.970	0.917

MAE, mean absolute error; RMSE, root mean square error; PSNR, peak signal noise ratio; SSIM, structural similarity; UQI, universal quality index.

### 4.2 Evaluation of prediction by different methods

Through the above analysis, it is found that the quality of prediction results of SPFNet is better, which may be due to the fact that we compress the connected feature graphs through global average pooling, simplifying the original fusion features along the spatial dimension clues, obtaining the global distribution of channel responses, selectively enhancing more information features, and suppressing less useful features. Additionally, in this work, to further validate the performance of the proposed model, we compare the SPFNet of MLGMF with other state-of-the-art DL methods, including Swin-UNet, TransQA, and IVPSQA. **Table 3** shows the quantitative results of 40 test cases, it is evident that MLGMF exhibits significant improvements over the previous three methods. Compared to Swin-UNet, TransQA, and IVPSQA in terms of MAE, it decreases by 59.8%, 5.6%, and 16.9%. Regarding SSIM, it improves by 2.1%, 0.8%, and 1.9% respectively. In terms of RMSE, it reduces by 64.2%, 18.3%, and 46.7% respectively.

**Table 3:** Comparison of experiments based on MLGMF and other prediction network models.

	MAE	RMSE	PSNR	SSIM	UQI
Swin-UNet	51.476	7.411	51.901	0.968	0.860
TransQA	34.012	5.342	51.721	0.981	0.910
IVPSQA	37.675	6.623	52.026	0.974	0.894
MLGMF	32.221	4.516	53.032	0.989	0.931

MAE, mean absolute error; RMSE, root mean square error; PSNR, peak signal noise ratio; SSIM, structural similarity; UQI, universal quality index.

**Figure 6:**
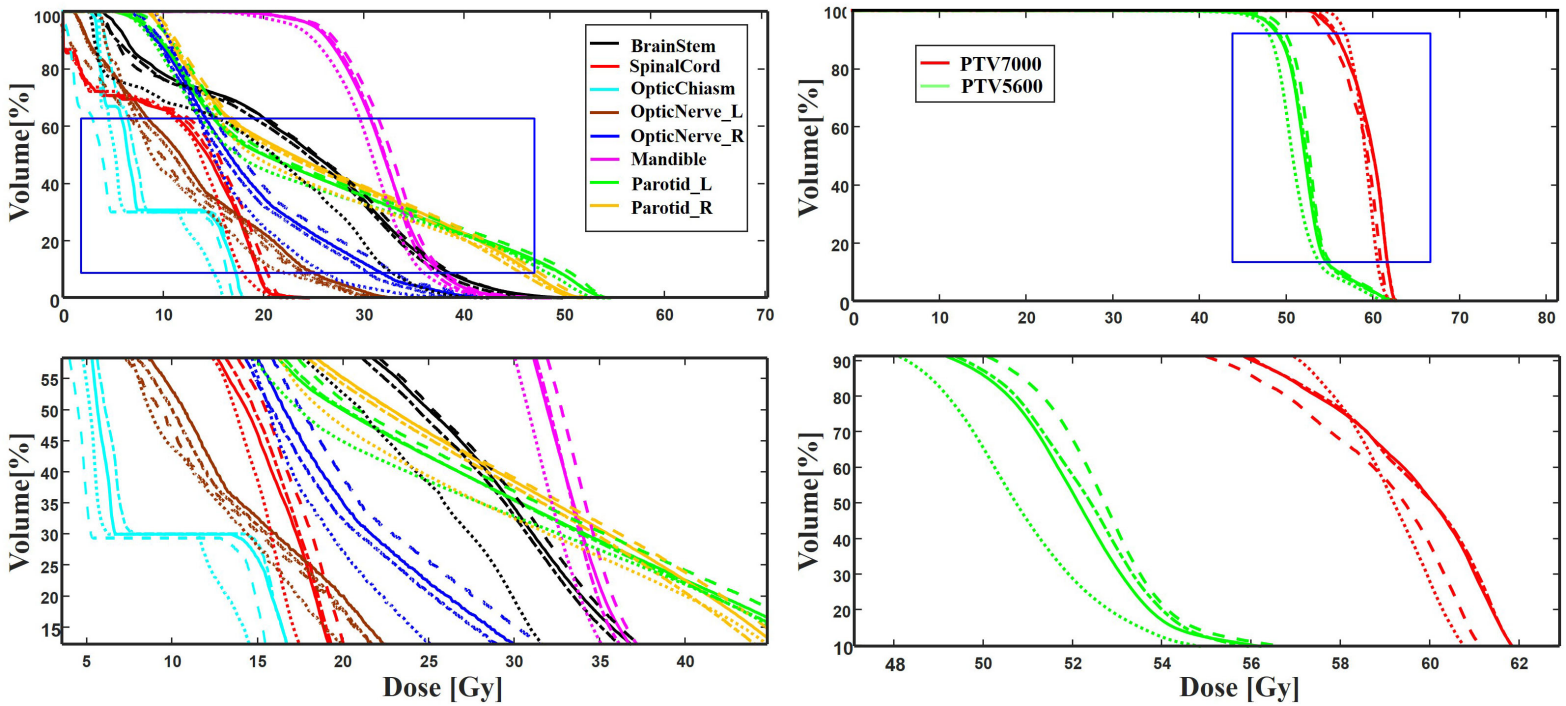
**A** typical DVH comparison for an example patient in the test dataset among ground truth (solid line), IVPSQA (dash line), TransQA (dotted line), and MLGMF (dash dot line). The second row is the zoomed details of the ROIs as indicated by the blue solid square in the first row.

**Table 4** summarizes the quantitative results for clinical interested metrics for PTVs and 17 OARs. The values of D2, D50, D98, V95, V100, Dmean, and Dmax values are reported for the PTVs, while Dmean and Dmax are reported for the OARs. In regard to the sparing of OARs, with the exception of Dmax for the InnerEar_R and Eye_R, we note that the predicted prePSQA metrics are in good agreement with the MDose. The proposed MLGMF model achieved the smallest errors for these targets and OARs, followed by TransQA and IVPSQA, Swin-UNet has the worst, and IVPSQA results were extremely close to TransQA. **Figure 6** shows a typical DVHs comparison for an example patient. Since the proposed models can account for multi-scale fusion features extracted from CT and RTDose through the incorporation of the MLGMF module into a multi-channel CNN, it is expected that the prediction of spatially related DVHs curves can also benefit from voxel-level-based PDose, as shown in the figure, where MLGMF is more consistent with GT values. The DVHs curves for IVPSQA and TransQA show some variation among the three approaches, and the predicted results have approximately identical shapes to that of the GT. Overall, the DVHs lines for all PTVs and OARs show good consistency and comparable performance with the GT.

**Table 4:** Quantitative evaluation based on the error in five model predictions of DVH metrics for targets and OARs.

Structure	Metric	Swin-UNet	TransQA	IVPSQA	MLGMF
PTV7000	Dmean	0.52 ± 0.67	0.19 ± 0.69	0.37 ± 0.53	0.11 ± 0.35
	D2	0.77 ± 0.34	0.26 ± 0.86	0.28 ± 0.63	0.20 ± 0.18
	D50	0.47 ± 0.94	0.16 ± 0.83	0.36 ± 0.52	0.14 ± 0.24
	D98	0.91 ± 1.51	0.34 ± 1.39	0.47 ± 1.97	0.12 ± 1.60
	V95	0.97 ± 0.45	0.73 ± 0.67	0.91 ± 0.14	0.46 ± 0.69
	V100	0.66 ± 1.70	0.46 ± 1.76	0.48 ± 1.01	0.37 ± 1.46
PTV5600	Dmean	1.10 ± 0.99	0.58 ± 1.02	0.69 ± 1.34	0.21 ± 1.47
	D2	0.52 ± 1.50	0.24 ± 1.50	0.42 ± 1.95	0.11 ± 1.86
	D50	0.86 ± 1.25	0.42 ± 1.02	0.69 ± 1.15	0.25 ± 1.08
	D98	0.88 ± 0.66	0.39 ± 1.05	0.45 ± 0.76	0.15 ± 0.54
	V95	0.94 ± 0.61	0.78 ± 0.42	0.82 ± 0.28	0.61 ± 0.25
	V100	1.82 ± 1.23	1.63 ± 1.36	1.66 ± 1.03	1.26 ± 1.36
Parotid_L	Dmean	3.20 ± 1.79	1.45 ± 1.75	1.50 ± 1.72	1.23 ± 1.16
	D50	1.86 ± 1.90	1.46 ± 1.99	1.53 ± 1.50	1.35 ± 1.46
Parotid_R	Dmean	2.26 ± 2.75	1.23 ± 1.14	1.31 ± 2.13	1.09 ± 2.68
	D50	2.71 ± 2.45	1.03 ± 2.55	1.07 ± 2.91	1.02 ± 2.95
OpticChiasm	Dmax	3.08 ± 1.30	1.28 ± 1.31	1.66 ± 1.64	1.19 ± 1.45
OpticNerve_L	Dmax	1.99 ± 1.29	1.13 ± 1.89	1.35 ± 1.66	1.12 ± 7.47
OpticNerve_R	Dmax	2.39 ± 1.43	1.36 ± 1.68	1.42 ± 2.54	1.00 ± 1.61
InnerEar_R	Dmax	2.58 ± 2.59	2.01 ± 2.95	2.37 ± 3.01	1.93 ± 2.36
InnerEar_L	Dmax	2.55 ± 0.87	1.21 ± 1.31	1.39 ± 1.53	1.10 ± 0.94
BrainStem	Dmax	1.93 ± 2.00	1.30 ± 1.96	1.60 ± 2.26	1.05 ± 1.33
SpinalCord	Dmax	1.73 ± 1.39	1.24 ± 1.23	1.56 ± 0.29	1.20 ± 1.01
Eye_L	Dmax	2.70 ± 1.31	2.17 ± 2.34	2.46 ± 0.57	1.80 ± 1.72
Eye_R	Dmax	2.59 ± 1.53	2.49 ± 2.56	2.50 ± 0.99	2.38 ± 2.17
Lens_L	Dmax	1.43 ± 1.62	0.32 ± 1.74	0.59 ± 0.26	0.27 ± 1.94
Lens_R	Dmax	0.84 ± 0.16	0.36 ± 1.40	0.64 ± 1.38	0.20 ± 1.55
Pituitary	Dmax	1.87 ± 1.70	1.47 ± 1.77	1.55 ± 1.27	1.25 ± 1.46
Mandible	Dmax	2.95 ± 2.90	2.49 ± 2.13	2.86 ± 1.04	2.08 ± 3.64
TMJ_L	Dmax	1.99 ± 2.00	1.65 ± 2.25	1.86 ± 1.66	1.44 ± 2.29
TMJ_R	Dmax	2.59 ± 2.91	1.75 ± 1.19	2.36 ± 2.18	1.42 ± 1.54

**Figure 7:**
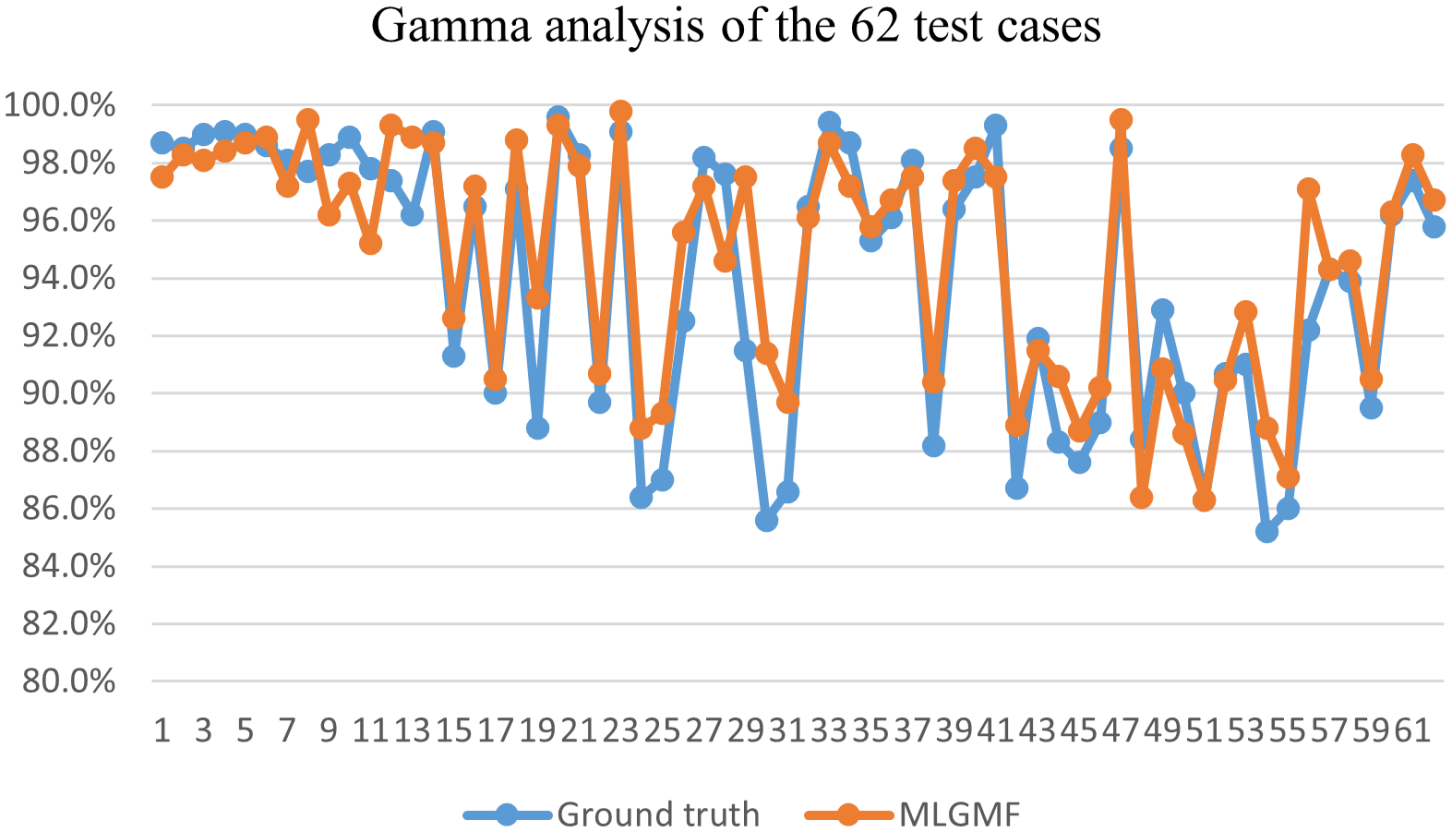
Gamma analysis of GT and MLGMF.

To verify the accuracy of these models, the PDose were compared using a 3%/3 mm global GPR analysis with a dose threshold of 10%. The GPR for the Swin-UNet model (95.4 ± 2.9) was inferior to the other methods, and the GPR for MLGMF (94.7 ± 3.9) was in good agreement with the GT (94.0 ± 4.6). The remaining methods, TransQA (95.1 ± 2.9), IVPSQA (95.2 ± 2.9), yielded comparable results. **Figure 7** plots the GPRs for the MDose and the predictions of MLGMF. If the accuracy of a prediction model is perfect, the GPR points for the test cases should be consistent with the GT. The mean errors between the GPR of the MDose and the predictions were 3.41% for the Swin-UNet, and 2.84%, 2.31%, 1.81% for IVPSQA, TransQA and MLGMF, respectively.

## 5 Discussion and conclusion

AI requires the use of complex mathematical algorithms that mimic human intelligence to perform tasks such as pattern recognition, visual perception and decision making. Recent technological advances have made RT highly sophisticated, with system equipment almost entirely depends on human-computer interaction. The increasing complexity of these human-computer interactions, coupled with rising cancer mortality, has led to a huge workforce shortage worldwide. PrePSQA involves an evaluation of the treatment plan and dose in order to detect human errors and potential anomalies in the calculation of the dose and plan delivery. Most treatment plans are subject to manual QA checks, and in rare cases these fail, meaning that many underlying factors need to be investigated and treatment for the cancer patient will be deferred. If an innovative algorithm can be designed to predict PRF or identify possible sources of error, it would be a valuable replacement for repetitive and labor-intensive tasks, and could eliminate the current reliance on manual measurements. However, most existing prePSQA prediction networks stack successive convolutional and pooling layers to obtain robust feature representations. This multiple pooling operation greatly reduces the spatial resolution of the features, and for deeper-level features, this causes a significant loss of local spatial detail. CNN-based approaches generally have limitations in terms of modeling explicit long-range relations, due to the intrinsic locality of convolution operations. So when faced with more difficult tasks, the desired outcome is often not obtained. This is especially true for target structures that show large inter-patient variations in terms of texture, shape, and size. In this work, we have presented a novel MLGMF module to achieve high-quality voxel-wise PDose values from whole-volume CT and RTDose images. A simple yet effective multi-level fusion operation was adopted in the proposed model to overcome the need for excessive convolutional and pooling operations in sequential learning with a CNN. Our experiment results indicate that the proposed model achieved comparable or better performance on MDose prediction, spatial dose differential distribution, DVHs of OARs/targets and GPRs compared to the existing state-of-the-art (UNet-based, Transformer-based, Invertible Neural network-based) methods.

An evaluation of DVHs between TPS and MDose yields deep insight into the dose distribution delivered to structures of interest; nevertheless, it has also reduced the efficiency of prePSQA due to the increase in the number of DVH metrics and treatment plans that needed to be reevaluated. Recently, by incorporating the plan information (MLC position, gantry/collimator angles, and monitor units) recorded in a machine log file into a vendor-provided calculation tool, the computational-based virtual prePSQA (VQA) has been used to perform a secondary check of the TPS dose. Various efforts with different capabilities on LINAC log file analysis have been proposed to improve the efficiency of VQA (33–38). In addition, the latest commercial calculation software (Mobius3D; Varian Medical Systems, Palo Alto, CA, USA) is available for clinical dose verification (39). Although the aforementioned studies indicate that VQA can provide an opportunity for evaluating TPS plans and possible for resource sparing, this is still an indirect descriptive method which is performed without practical measurements on a physical phantom, and might not be able to detect errors in MLC positions due to T-nut or motor failures. The MDose-based MLGMF suggested in this study strikes a nice balance between DVHs metric and efficiency. Compared with other comparative models, relatively low dose errors were discovered between the PDose of MLGMF and GT (**Figures 4**-**6**). The results of quantitative analysis are shown in **Tables 2**-**4**, which further proves the accuracy of the proposed models.

Research on direct prePSQA dose prediction from 3D measured dose for AI models is crucial, yet rare. Almost all prePSQA prediction methods use 2D or 1D data or a combination of both as inputs for predicting GPRs or identifying potential errors (11–22). Using only this information as input for prePSQA prediction may cause a loss of spatial information about the complex dose distribution, and can limit the model’s predictive ability. Yue et al. proposed a DL method of dose prediction for nasopharyngeal carcinoma by taking advantage of multiple sources of information, such as distance information, binary mask information of OARs/targets, planning CT, and clinical plans (40). They achieved values for the predicted dose error and DVHs error that were 7.51% and 11.6% lower than those of the mask-based method, respectively. Hirashima et al. and Tomori et al. also found that combining multiple sources of input information could improve the prediction and classification performance (17, 19, 20). Recently, a new DVHs-based prePSQA prediction methodology was developed by combining DL and ML techniques. This prediction model achieved values for the area under curve, accuracy, sensitivity, and specificity of 0.89 versus 0.88, 0.89 versus 0.86, 0.71 versus 0.71, and 0.95 versus 0.91 for the measured and predicted doses, respectively, indicating that this method was promising in terms of overcoming the limitations of GIs and improving the efficiency of IMRT/VMAT delivery (31). With the aim of fully exploiting the mutual promotion between the different modalities, we proposed the MLGMF module to extract multi-scale features which are specific to the CT and RTDose modalities. Unlike in previous studies, features from both the whole-volume dose branch and the CT branch were fused in each fusion module of our network.

For the PDose distributions shown in **Figure 4**, our proposed models showed better performance, and the ResUNet model performed worse. An interesting result was that most of the values predicted by CCFNet and SCFNet were lower than the GT, resulting in large negative areas in their difference maps, especially for SCFNet. However, CPFNet and SPFNet showed the opposite phenomenon. One possible reason for this is that the four models differ in terms of the final feature fusion operation. To further enhance the mutual interaction between the CT and RTDose modalities, we replaced the CPFNet and SPFNet with cross-fusion concatenation in the last stage. In this way, the mutual advantages between different modalities could be further explored. From **Table 3**, we compare our model with Swin-UNet, TransQA, and IVPSQA methods, the quantitative metrics show that MLGMF exhibits significant improvements over the previous three methods, which means relatively low dose errors were discovered between the PDose of MLGMF and GT. With regard to DVHs metrics evaluation, Gronberg et al. reported that their predicted DVHs metrics for the Dmax and Dmean of the OARs were within 2.7% and 2.0% (41). Nguyen et al. achieved overall mean values for all OARs of within 5.1% of the prescription dose for Dmean (42). Zhang et al. reported average voxel-based MAEs (normalized to the prescription dose) of within 6.9% for all structures (43). In comparison, our model achieved DVH metrics for Dmax and Dmean for all OARs of within 2.4% and 1.3% (**Table 4**). More specifically, compared with these methods studied for auto-planning purposes by using only CT or RT dose as inputs, our modules were trained based on the anatomical features extracted from CT images and the dosimetric features from the MDose and RTDose. The proposed models were able to operate directly on the dose modalities to learn and extract their own spatial features, and to improve the quality of representation for the further generation of channel-wise features. Hence, our models achieved similar or even better prediction accuracy on DVHs metrics compared with the alternatives for H&N VMAT cases. In addition, we observed that the deviations between the predicted and measured GPRs were mostly within 6%. Recent publications support the use of plan complexity or LINAC performance metrics to predict GPR for IMRT/VMAT plans. Since most samples in the input data have GPR values larger than 90%, and only a tiny fraction of samples has GPRs lower than 90%, the unbalanced GPR data distribution could result in large prediction errors. The proposed prePSQA approach can generate robust GPR outcomes from high-quality PDose results, which perfectly avoids the problem of data imbalance.

DL networks have good learning ability and can predict better dose distribution through prior knowledge. Although our method has advantages over other methods, DL networks still have some shortcomings in dose prediction. It was found in the experiment that all four models in this paper predicted some unpredictable dose points, and this uncertainty deserves further analysis. We considered whether more constraint factors could be introduced in the training process to solve this problem. There are several potential limitations to this study. Firstly, the use of CT and RTDose distributions as input information for the proposed model may limit its predictive ability to some extent. Combining multiple sources of information, such as target/OARs structures, treatment plans and LINAC performance-related metrics, may increase the predictive accuracy of the prePSQA model. The addition of more relevant information to the input of the model in order to achieve higher accuracy with fewer labor costs will be a topic for future research. Secondly, although we enrolled 310 H&N cases with 620 VMAT arcs, IMRT, TOMO, etc. are widely used RT method for H&N cases, due to the inherent requirements of the model training, the number of patients was relatively small, and our conclusions could be further supported by increasing the sample size and the multimodal dataset in future studies. Last but not least, this work focused on using a DL-based method to solve the prePSQA problem, rather than the network itself. In the future, we hope to refine the MLGMF approach to achieve higher precision accuracy. Nevertheless, the actual delivered dose distribution based PDose will be a breakthrough in predicting prePSQA, in combination with the novel multi-level gated modality fusion networks, the new prePSQA framework is capable of driving improvements in RT and may become better than current or past clinical practice. AI methods have shown good results in radiotherapy dose prediction, but it is not yet appropriate to directly use the measurement dose prediction and replace manual measurement. After further improvements to our approach, it may be a great help for adaptive RT and play a considerable role in future prePSQA work.

## Data Availability

The raw data supporting the conclusions of this article will be made available by the authors, without undue reservation.
